# Aspirin-Exacerbated Respiratory Disease Requiring Desensitization Prior to Planned Percutaneous Catheterization Intervention

**DOI:** 10.7759/cureus.26686

**Published:** 2022-07-09

**Authors:** Eric Landa, Saad Javaid, Erika Vigandt, Frederick Campos, Luis Mercado

**Affiliations:** 1 Internal Medicine, Unity Health, Searcy, USA; 2 Internal Medicine, Wyckoff Heights Medical Center, Brooklyn, USA; 3 Internal Medicine, The Brooklyn Hospital Center, Brooklyn, USA

**Keywords:** nsaid, pci, aspirin, desensitization, aerd

## Abstract

Aspirin-exacerbated respiratory disease (AERD) consists of a triad of asthma, chronic rhinosinusitis with nasal polyposis, and a hypersensitivity reaction to aspirin consisting of nasal congestion and broncho-constriction. This disease presents a conundrum in cardiac patients undergoing percutaneous catheterization intervention (PCI) who might require stent deployment due to the need for aspirin as part of the dual antiplatelet therapy required if a stent is placed. Here, we present the case of a patient who underwent a coronary angiogram showing two-vessel disease but had to undergo aspirin desensitization first before planned PCI as he had a history of severe aspirin allergy in the past.

## Introduction

Aspirin allergy is found in approximately 7% of individuals with asthma, with the majority also allergic to one or more non-steroidal anti-inflammatory drugs (NSAIDs) [1]. Allergy to aspirin was first described in 1902 shortly after its introduction. The triad of asthma, chronic rhinosinusitis with nasal polyposis, and a hypersensitivity reaction to aspirin, also known as Samter’s triad, was described in 1968 [2]. Aspirin or NSAID hypersensitivity can be divided into six different categories depending on the eliciting NSAID, signs and symptoms, the timing of onset of symptoms, and comorbidities. Typical symptoms observed include urticaria, angioedema, and respiratory symptoms such as rhinitis, nasal obstruction, and bronchospasm. Patients undergoing percutaneous catheterization intervention (PCI) with stent placement require dual antiplatelet therapy (DAPT) which includes lifelong daily aspirin use. This becomes a problem in patients with allergic reactions to aspirin and thus requires desensitization. This case report discusses a patient with aspirin-exacerbated respiratory disorder (AERD) requiring desensitization.

## Case presentation

**Figure 1:**
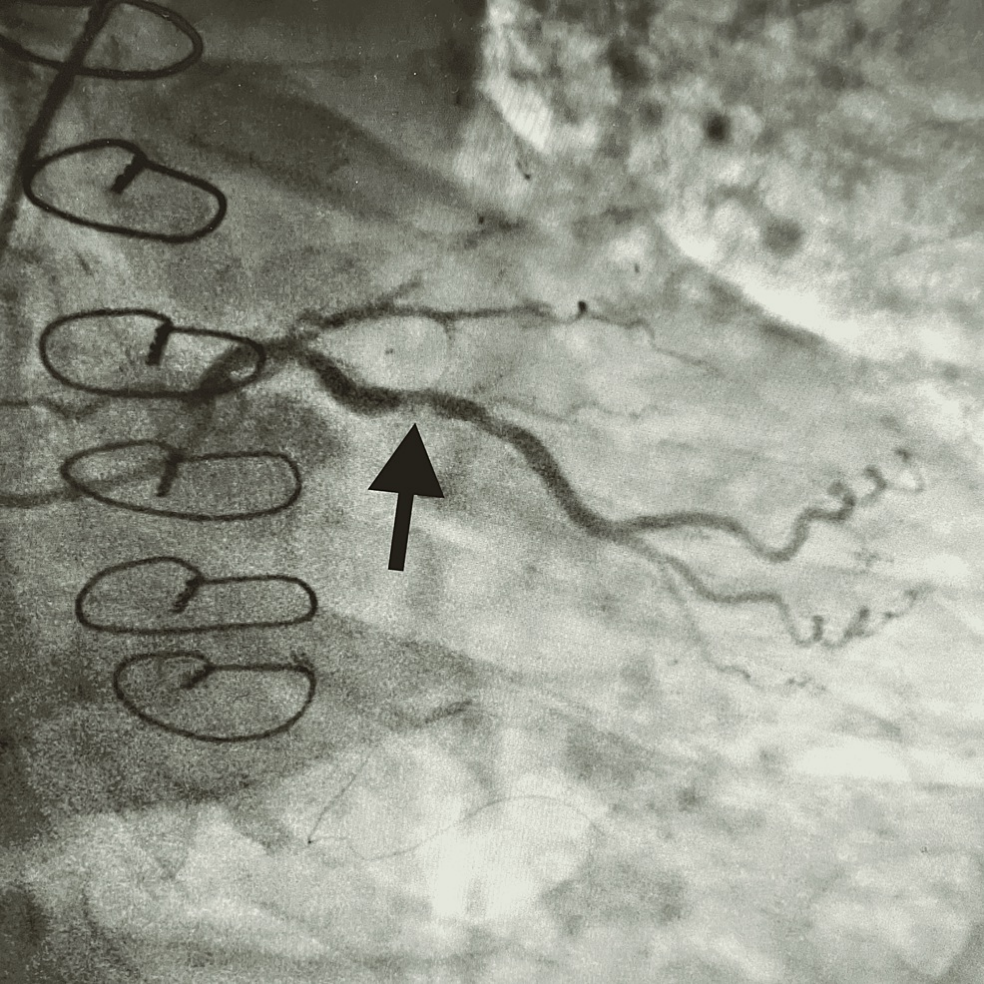
Occlusion of the left circumflex artery.

A 67-year-old male with a medical history significant for coronary artery disease (CAD) post-coronary artery bypass graft (CABG) in 2016, primary hypertension, diabetes mellitus type II, asthma, and hyperlipidemia presented to the hospital complaining of left-sided chest pain that began while he was in the shower that morning. He described the pain as sudden in onset, pressure-like, non-radiating, worsened with movement and deep inspiration, and nothing made the pain better. The pain was constant and lasted for about two hours. He stated that, usually, he was able to walk five blocks and climb three flights of stairs before getting short of breath. He slept with two pillows at night. He denied any fever, chills, headache, shortness of breath, palpitations, abdominal pain, or changes in bowel or bladder habits. He was a former smoker with an unknown number of packs per day but had quit six years ago. On arrival, his vital signs were as follows: temperature of 98.7°F, blood pressure of 132/64 mmHg, heart rate of 62 beats/minute, and oxygen saturation of 98%. Laboratory results were significant for negative troponin levels, and the rest of the labs were unremarkable. The electrocardiogram showed normal sinus rhythm and no ST-T-wave abnormalities. He had a HEART score of 6 and a Thrombolysis in Myocardial Infarction (TIMI) score of 3. An echocardiogram performed showed left ventricular hypertrophy, an ejection fraction of 55-60%, and wall motion abnormalities. The cardiology team was consulted, and after evaluation, a coronary angiogram was performed which revealed 40% stenosis of the left main artery, 100% stenosis of the proximal left anterior descending artery (LAD), 85% stenosis of the circumflex artery, 80% stenosis of the proximal right coronary artery (RCA), and 100% stenosis of the right posterior descending artery (PDA) (Figure 1).

**Figure 2:**
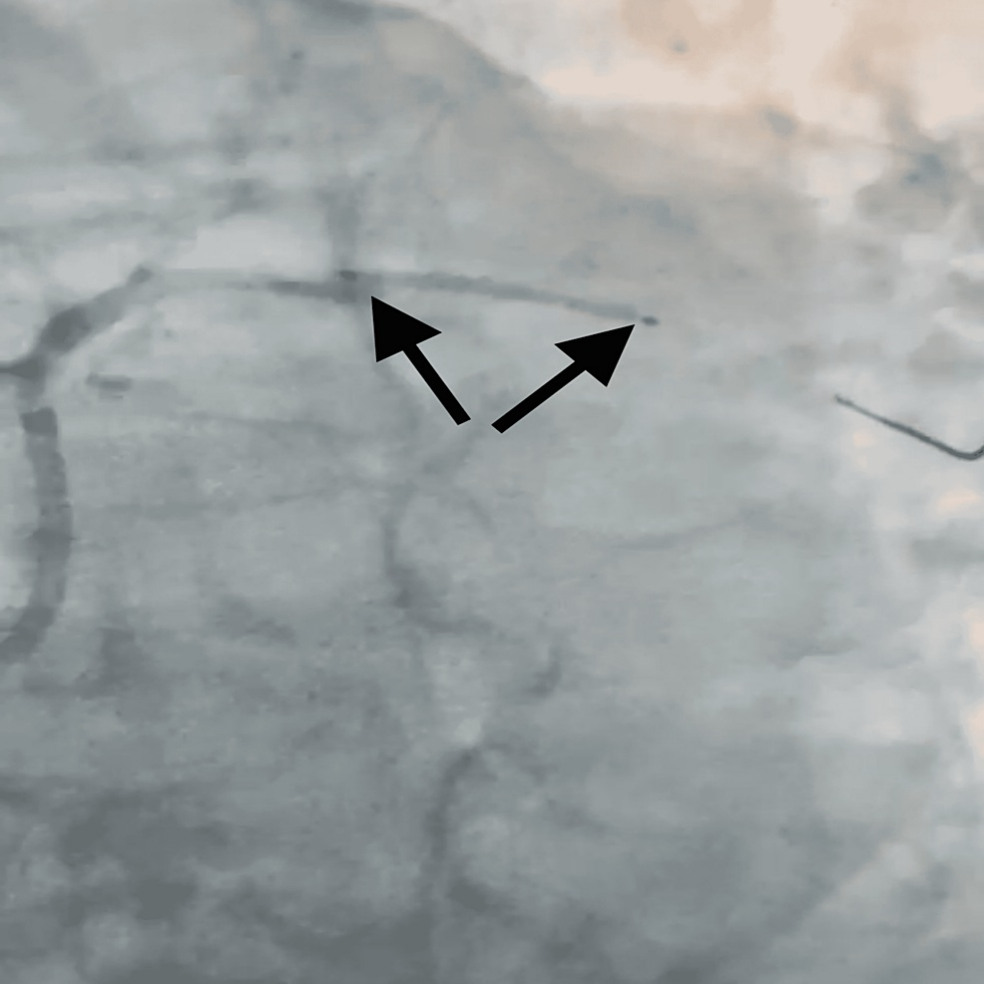
Stent placement in the left circumflex artery.

Given these findings, a PCI was planned; however, given the patient’s history of a severe allergic reaction to aspirin, after he underwent CABG during which he developed hives and angioedema, aspirin desensitization was planned. The patient was transferred to the intensive care unit (ICU) for close monitoring during the desensitization and PCI was planned for two days later. While in the ICU, on further history taking, the patient stated that he had chronic rhinosinusitis, and, upon examination, he was noted to have a right nasal polyp. He met the criteria and was diagnosed with AERD. Aspirin desensitization was performed as per hospital guidelines and lasted for a total of four hours during which aspirin was administered in increasing amounts with a 30-minute interval in between each dose for a total of seven doses which was equivalent to approximately 365 mg of Aspirin. The patient tolerated the process well without any allergic reactions. The next morning he was taken for PCI where he received one stent to the obtuse marginal artery 1 (OM1) and one stent in the proximal circumflex artery (Figure 2).

He was scheduled for a staged PCI of the RCA in four weeks. He was sent home on aspirin 81 mg daily and clopidogrel 75 mg daily along with other medications. He was followed up in the clinic one week later and was doing well, denied any more episodes of chest pain, and had no complaints.

## Discussion

AERD is characterized by the combination of asthma, chronic rhinosinusitis with polyps, and the upper and lower respiratory tract symptoms caused after the ingestion of aspirin and other cyclooxygenase 1 (COX-1) inhibitors, and NSAIDs. The term AERD focuses more on the underlying chronic upper and lower respiratory tract infections exacerbated after exposure to NSAIDs [3,4]. The term pseudoallergic reaction is used to describe allergic reactions after exposure to aspirin as there is no involvement of the immunoglobulin E-mediated antibody reactions. On the contrary, abnormal biochemical response to the pharmacologic actions of NSAIDs is the underlying mechanism responsible for the exacerbations and airway manifestations. The prevalence of AERD varies depending upon the underlying conditions and their severity. Patients with mild-to-moderate asthma have a prevalence of 7%, while patients with severe asthma are believed to have a prevalence of 14% [5]. Similarly, the prevalence is about 10% and 9%, respectively, among patients with nasal polyposis or chronic rhinosinusitis as the predominant manifestations.

The pathophysiology of AERD is not widely understood and is multifactorial. It is believed to be caused by the dysregulation of arachidonic acid (AA) metabolism, which results in the overproduction of leukotrienes. Prostaglandin E2 (PGE2), which acts as a brake on leukotriene production, is dysregulated and causes unopposed activation of the 5-lipoxygenase (5-LO) pathway. COX-1 inhibitors (which block the production of PGE2) cause further exacerbation of leukotriene production [6-10]. The bronchial mast cells and eosinophils are considered the primary source of leukotriene production in AERD, and exposure to aspirin and other mast cells causes upregulation and increased manifestation of these cells. Moreover, this upregulation causes overexpression of the enzyme leukotriene C4 (LTC4) synthase, which mediates the formation of LTC4. Similarly, one of the prostaglandins, prostaglandin D2 (PGD2), which has a predominantly bronchoconstrictor effect, is also overproduced in AERD and is further increased after the aspirin challenge test [9,10].

The clinical manifestations vary among the patient population, and, as mentioned above, patients develop asthma and chronic rhinosinusitis with polyps, and it is associated with exacerbations after exposure to aspirin and other NSAIDs. The symptoms usually develop in adulthood and progress over the years. Compared to aspirin-tolerant asthma, patients with AERD have more severe asthma and are frequently intubated with increased requirements of systemic steroids for symptom resolution. Similarly, patients develop refractory rhinosinusitis that is not responsive to the usual treatment and results in mucosal hypertrophy, anosmia, nasal polyposis, and frequent congestions. In a survey of 190 patients, it was reported that in patients with AERD, the quality of life was severely affected, and the major contributors to this were chronic recurrent rhinosinusitis and the nasal symptoms associated with anosmia [11].

The reaction to NSAIDs develops in 30 minutes to three hours after ingestion, peaks in one to two hours, and resolves in three to four hours after onset [12,13]. Smaller doses produce less severe symptoms, with larger doses causing more severe and life-threatening symptoms leading to hospitalizations and recurrent intubations. Most reactions occur with low doses of 30-120 mg of aspirin, and very few require higher doses. Similarly, equivalent doses of other NSAIDs cause a similar chain of reactions. The upper respiratory tract symptoms mainly involve nasal congestion/obstruction, periorbital edema, injection of the conjunctiva, and/or watery rhinorrhea [13]. The lower respiratory tract symptoms are the hallmark of asthma associated with AERD and consist of wheezing, dyspnea, cough, and chest tightness. These symptoms are usually reversible with bronchodilators as the pathophysiologic mechanism associated with these symptoms is smooth muscle contraction causing a marked reduction in forced expiratory volume in one second (FEV1). Chronic exposure to triggers over a period of time causes the symptoms to develop more frequently. The diagnosis of AERD is often clinical and is characterized by the presence of all the typical manifestations of asthma, chronic rhinosinusitis, and a clear history of exacerbations with NSAID exposure. The diagnosis becomes challenging when isolated symptoms are predominantly present. It is also important to distinguish adverse reactions to NSAIDs in patients with asthma and rhinosinusitis from NSAID hypersensitivity which is an acquired condition, especially because most of the population is not aware of these sensitivities [13,14]. As NSAID use is high in the population experiencing upper respiratory tract infections, a clear association is hard to establish as these conditions also serve as a trigger for asthma and related symptoms. The aspirin challenge test is the only valuable and definite diagnostic test to establish the association between the array of symptoms and NSAID exposure. Patients with NSAID sensitivity usually develop a reaction to a single NSAID use, and in that case, there is an 80% likelihood of having a positive oral aspirin challenge test. The likelihood of the test increases to 90% of patients with sensitivity to two NSAIDs [14]. The aspirin challenge test is usually performed orally, while intranasal or bronchial challenge can also be performed. Patients undergoing this testing are usually pretreated with leukotriene-modifying agents (LTMAs) as these medications reduce the severity of pulmonary reactions following the exposure. This testing is performed appropriately by pulmonary and allergy specialists. Various institutions have various guidelines for the oral aspirin challenge test, but it usually starts with a low dose and doubles every three to four hours [15]. The treatment of AERD depends on the severity and frequency of symptoms and begins with the avoidance of triggers and the medical and surgical management of chronic rhinosinusitis and lower respiratory tract symptoms. LTMAs are very vital in the management of AERD symptoms and are recommended as the initial therapy. These agents address the underlying leukotriene dysregulation and result in the resolution of symptoms.

Both leukotriene-receptor antagonists (LTRAs) (montelukast and zafirlukast) and inhibitors of leukotriene synthesis (zileuton) provide relief in AERD [15,16]. Similarly, asthma in AERD is treated based on national and international guidelines. Certain inflammatory conditions and atherosclerotic heart/vascular diseases necessitate the use of NSAIDs and antiplatelets; hence, the definite and ultimate treatment is aspirin desensitization. The mechanism of aspirin desensitization and its altered response to NSAIDs is not entirely understood, and it is believed that several mechanisms play an essential role. Inhibition of transcription factors, signal transducers, and activator of transcription 6 causes reduced expression of leukotrienes with daily exposure to aspirin during the desensitization [17,18]. Another mechanism is the drug’s ability to impair the production of PGD2 by mast cells, as PGD2 is pro-inflammatory and causes the recruitment of inflammatory cells. Though aspirin desensitization is a useful and cost-effective intervention, some patients develop reactions during the desensitization process and are not able to receive it. In such patients, monoclonal antibodies have proven to be efficacious and valuable.

## Conclusions

Aspirin allergy is commonly seen in patients with asthma; however, AERD is not encountered often and can be considered a somewhat rare diagnosis. Aspirin is one of the DAPT medications that is necessary after a coronary stent is placed to prevent occlusion of the stent. It is especially challenging when a patient requiring stent placement is allergic to aspirin as desensitization attempts may fail. Here, we presented the case of a patient with AERD who underwent successful desensitization and stent placement.
